# Chimeric hippocampal organotypic slices enable the *in vitro* investigation of human iPSC‐derived microglia in a three‐dimensional environment with preserved neural network activity

**DOI:** 10.1002/alz.088649

**Published:** 2025-01-03

**Authors:** Jens Devoght, Annelies Nonneman, Sofie Swijsen, Dirk Wuyts, Johanna Van den Daele, Hervé Maurin, Winnok H De Vos, Andreas Ebneth, Pei‐ Yu Shih, Juan Diego Pita Almenar

**Affiliations:** ^1^ Janssen Research & Development, A Division of Janssen Pharmaceutica, Beerse, Belgium, Beerse Belgium; ^2^ Charles River Laboratories, Beerse Belgium; ^3^ University of Antwerp, Wilrijk, Antwerp Belgium

## Abstract

**Background:**

Microglial cells have emerged as key players in the pathogenesis of Alzheimer’s disease (AD). They act as a first line defense and fulfil a crucial role during brain development and circuit homeostasis. Microglia are involved in the removal of debris, control neural activity, regulate synaptic plasticity, and synapse pruning. Normal microglial functioning can be impaired by aging, stress, and genetic predisposition, provoking a pathophysiological turnover towards excessive neuroinflammation, neurodegeneration and synaptic loss, all hallmarks of AD. Despite similarities between human and mouse microglia, major discrepancies exist across species regarding gene expression and functionality. Even more, a variety of human microglial risk genes lack the proper murine orthologues, urging for the use of human cell‐based assays. Although *in vitro* (human) cell culture systems provide useful insights of microglial functionality, they lack a functional three‐dimensional brain environment.

To investigate human microglia in a functional brain network, we developed an easy‐to‐manipulate chimeric platform of murine hippocampal organotypic slice cultures (OSCs) with integrated human iPSC‐derived microglia.

**Method:**

OSCs depleted from endogenous mouse microglia were engrafted with human iPSC‐derived microglia to develop a chimeric OSC model which was validated using electrophysiology, immunostaining, RT‐qPCR and MSD.

**Result:**

The intact neural network activity of OSCs and effects of microglial depletion and activation were verified using a high‐density multielectrode array. Further evaluation of endogenous microglial effects on synaptic signaling within OSCs was measured using whole‐cell patch‐clamp. A co‐immunostaining with STEM121 (human) and IBA1 (microglia) validated the engraftment and presence of mature human iPSC‐microglia. Morphological analysis of the engrafted human iPSC‐microglia revealed a larger cell body, a higher number of ramifications per microglia and an increase in length of these ramifications, when compared to monocultures. Besides validation of the expression of microglial markers and morphological assessment, proper functioning of these human iPSC‐microglia engrafted cells was confirmed. The engrafted human iPSC‐microglia revealed damage‐induced migratory responses and release of human‐specific proinflammatory cytokines upon LPS stimulation.

**Conclusion:**

The developed chimeric OSCs model with engrafted human iPSC‐microglia provides an *ex vivo* platform to investigate human iPSC‐derived microglia within a physiologically relevant and functional brain network.

